# Isolation and identification of halotolerant soil bacteria from coastal Patenga area

**DOI:** 10.1186/s13104-017-2855-7

**Published:** 2017-10-30

**Authors:** Shafkat Shamim Rahman, Romana Siddique, Nafisa Tabassum

**Affiliations:** 1 Biotechnology Program, Department of Mathematics and Natural Sciences, BRAC University, 66, Mohakhali, Dhaka, 1212 Bangladesh; 2 Present Address: United Surgical (BD) Ltd, Plot# 659-661, Islampur, Kadda, Gazipur, 1702 Bangladesh

**Keywords:** Halotolerant, *Enterobacteriaceae*, *Clostridium*, *Corynebacterium*, Salinity

## Abstract

**Objective:**

Halotolerant bacteria have multiple uses viz. fermentation with lesser sterility control and industrial production of bioplastics. Moreover, it may increase the crop productivity of coastal saline lands in Bangladesh by transferring the salt tolerant genes into the plants. The study focused on the isolation and identification of the halotolerant bacteria from three soil samples, collected from coastal Patenga area. The samples were inoculated in nutrient media containing a wide range of salt concentrations.

**Results:**

All the samples showed 2, 4 and 6% (w/v) salt tolerance. The isolates from Patenga soil (4, 6%) and beach soil (2%) showed catalase activity and all the isolates showed negative results for oxidase activity, indole production, lactose and motility. All the samples provided positive results for dextrose fermentation. Other tests provided mixed results. Based on the morphological characteristics, biochemical tests and ABIS software analysis the isolates fall within the *Enterobacteriaceae, Clostridium* and *Corynebacterium*, with a predominance of *Vibrio*s. Overall the isolates can be considered as mild halotolerant, with the best growth observed at lower salinities and no halophilism detected. Among many possibilities, the genes responsible for the salt tolerant trait in these species can be identified, extracted and inserted into the crop plants to form a transgenic plant to result in higher yield for the rest of the year.

## Introduction

Salinization is one of the root reasons for the crop destruction in Bangladesh [1, 2]. Almost 20% of the country is covered as the coastal area from which about 53% of lands are affected by very slight to very strong salinity [3]. The upward movement of the saline ground water in the dry season (November–May) is the factor that initiates the development of saline soil. Nevertheless, the intrusion of the seawater also increases the degree of salinity of the coastal drinking water and cause severe health problems viz. hypertension or high blood pressure, stroke, heart diseases, pre-eclampsia.

Bacteria that grow in the absence of salt and in the presence of high salt concentrations are known as halotolerant. Non-halotolerant which can grow in low salt concentration about 1% w/v. Slightly tolerant as *pseudomonads, enterobacteria*, and *vibrio*s, can survive in up to 2–8%, moderately tolerant 18–20% and extremely tolerant microbes can grow over the whole range of salt concentrations from zero to saturation. The halotolerant organisms maintain a low level of ionic concentrations to synthesize compatible solutes to balance the osmotic level inside the cytoplasm with the outer medium. These maintenance mechanisms of the internal environment and the properties of the cytoplasmic membrane help them to adapt to changes in the saline environment as salt lakes, saline soils, and salted food products [4].

It was reported that amelioration of salt stress inhibitory effect on the canola seed germination was attributed to the inoculation of ACC deaminase-producing halotolerant bacteria modulating ethylene emission and inducing hydrolytic enzymes [5]. A research was executed on plant growth-promoting rhizobacteria (PGPR) containing aminocyclopropane-1-carboxylate (ACC) deaminase, [6] examined their effect on salinity stress tolerance in okra through the induction of ROS-scavenging enzyme activity. PGPR inoculated plants exhibited higher germination percentage, growth parameters, and chlorophyll content than control.

In a salt stress improvement research for red pepper plants by 1-aminocyclopropane-1-carboxylic acid (ACC) deaminase producing halotolerant bacteria was studied [7, 8]. The result showed salt stress ethylene production by increasing enzyme activities of a biosynthetic pathway. It was also reported that the growth promotion in inoculated red pepper plants under inhibitory levels of salt stress is due to ACC deaminase activity present in the halotolerant bacteria [9].

Halophilic and halotolerant bacteria are essential for salty foods production as Thai fish sauce, pickling brines and salt-cured bacon [10]. Isolates from effluents of textile industries also showed the ability to decolorize the utilized azo dyes [11]. Halotolerant bacteria, recovered from the composting process, were able to produce hydrolases, lipases, proteases, amylases, cellulases and biopolymers [12]. Four halotolerant species (*Bacillus atrophaeus, Halomonas shengliensis, Halomonas koreensis* and *Virgibacillus salarius*) showed the ability to metabolize hydrocarbons and isolates as *V. salarius* and *Brevibacillus* sp. KUMAs1 has the potential to be used for bioremediation [13, 14]. Nevertheless, *Corynebacterium xerosis* was the potent degraders of hydrocarbons (petrol and diesel) [15]. Halophilic and halotolerant bacteria can be used for the production of enzymes with different immunological properties [16] and also essential for nutrient recycling and for maintaining the soil health in a salty environment [17].

The goal of the research was to isolate and identify halotolerant bacteria from natural sources [18]. Multiple uses of these species viz. fermentation with lesser control on sterility and industrial production of bioplastics, can be beneficial for different sectors in Bangladesh. Besides that, it may provide the salt tolerant genes for plants in future. Therefore, plants can uptake and store salt as a nutrient and result in a good yield throughout the year.

## Main text

### Methods

#### Sample collection

Three different soil samples (Patenga Beach soil 22°15′06.8″N, 91°45′29.5″E; Land soil 22°14′31.6″N, 91°47′15.9″E and Patenga area soil 22°14′25.4″N, 91°48′59.7″E) were collected from 10 to 12-in. depth at different locations of the coastal Chittagong area; these were kept in a sterile polythene packet at room temperature.

#### Isolation and screening

5 g of each of the soil samples were taken to prepare a suspension. Then serially diluted (10^−3^, 10^−5^ and 10^−7^, 100 μL) samples were taken and incubated on the nutrient agar plates containing 2% (w/v), 4% (w/v), 6% (w/v), 8% (w/v) and 10% (w/v) NaCl for 24-h at 37 °C. No bacterial growth observed for 8% (w/v) and 10% (w/v) NaCl plates.

#### Biochemical tests

Total 18 primarily screened isolates (2 each from 2, 4 and 6% plates) were sub-cultured on NA plates without salt with the same dilution factor and nine inocula were tested further. After 24-h of incubation at 37 °C, the plates were observed [19].

Standard gram staining protocols were followed and then slide observed under a microscope. The presence or absence of bubbles or foam was observed to determine the capability of catalase activity [20]. Two drops of oxidase reagent *p*-aminodimethylaniline oxalate were added to the surface of test organisms’ growth in oxidase test [21]. Nitrate broth (beef extract 3 g/L, peptone 5 g/L, potassium nitrate 5 g/L) [22] and MR-VP broth (peptone, dextrose and potassium phosphate) was prepared and incubated at 24-h at 37 °C for Methyl Red test and VP test [23]. The triple sugar iodine agar was prepared and after inoculation, incubated for about 24-h at 35 °C for TSI test [24]. After inoculation, MIU media (MIU test [25]) and Simmon’s agar slants were also incubated for 24-h at 37 °C [22, 23]. Trypticase 10 g/L, NaCl 5 g/L, phenol red 0.018/L, sugar (glucose, lactose or sucrose) 5 g/L contained broth incubated at 37 °C for 24-h to determine the capability of fermenting carbohydrate substrate with acid and gas production [26]. Autoclaved starch agar (beef extract 3 g/L, soluble starch 10 g/L and agar 15 g/L) plates were incubated for 24-h to indicate the presence of α-amylase [27]. Sterile tubes containing inoculum was transferred in 6.5% NaCl solution and kept in 24-h incubation to assess salt hindrance.

### Results

All the samples showed growth in NA media containing 2% (w/v), 4% (w/v) and 6% (w/v) NaCl. But failed to grow in media containing 8% (w/v) and 10% (w/v) NaCl (Table 1).

**Table 1:** Results of growth in NA media containing 2% (w/v) to 10% (w/v) NaCl

Conc.	Patenga soil					Beach soil					Land soil				
	2%	4%	6%	8%	10%	2%	4%	6%	8%	10%	2%	4%	6%	8%	10%
Growth	✓	✓	✓	x	x	✓	✓	✓	x	x	✓	✓	✓	x	x

**Fig. 1:**
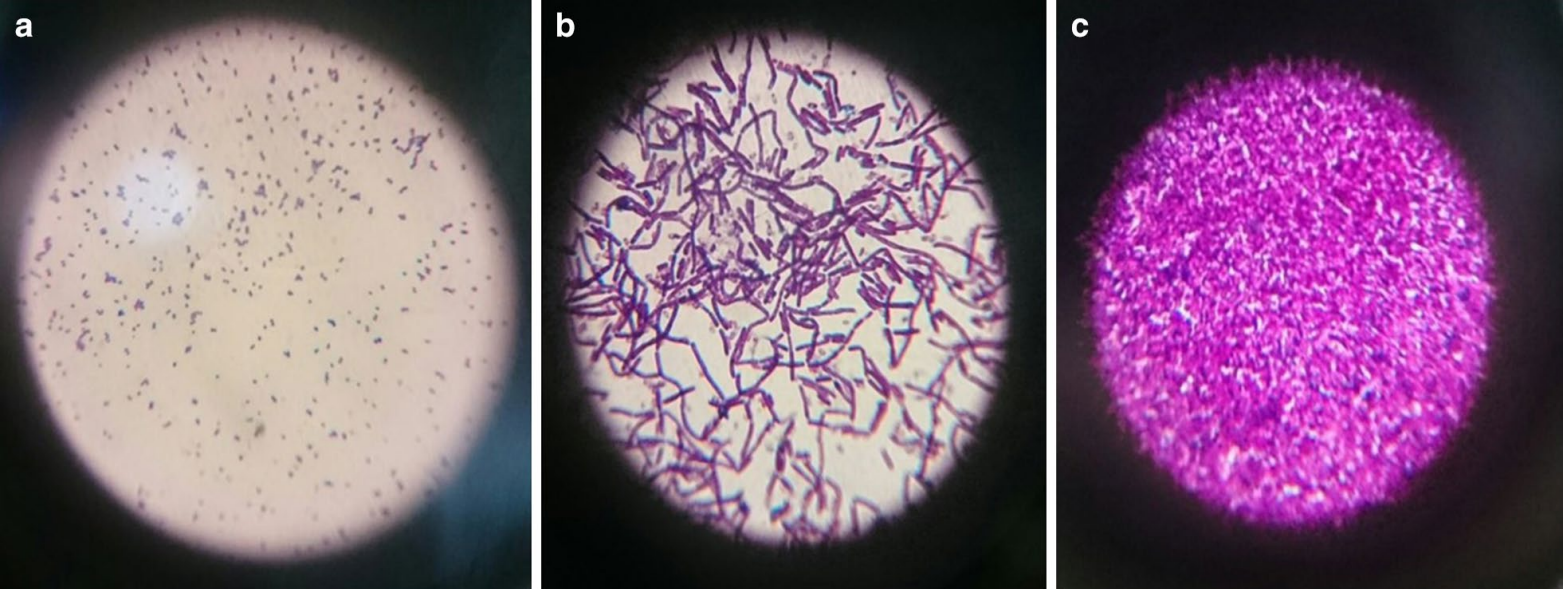
Gram positive bacterial isolates from **a** Patenga area soil, **b** Beach soil and **c** Land soil sample

The isolates were all gram positive (Fig. 1; Table 2). Patenga 4, 6% were cocci shaped and rest were rod shaped.

**Table 2:** Results of biochemical and sugar tests of the isolates collected from nutrient agar

Isolate no	Sample isolate name	Gram stain		TSI				MIU			Catalase	Oxidase	Simmon’s citrate	MR	VP	Nitrate reduction	Starch hydrolysis	Carbohydrate			6.5% NaCl solution	Presumptive organism
		±	Shape	BUTT	SLANT	H_2_S	Gas	Motility	Indole	Urease								Sucrose	Lactose	Dextrose		
1.	Patenga area soil 2%	+	Rod	A	A	−	−	−	−	−	+	−	−	+	−	−	−	+	−	+	+	*Brevibacillus agri*
2.	Patenga area soil 4%	+	Cocci	A	K	−	−	−	−	−	−	−	−	−	+	−	+	+	−	+	+	*Pantoea stewartii* subsp. *stewartii*
3.	Patenga area soil 6%	+	Cocci	K	A	−	−	−	−	+	+	−	−	−	+	−	−	+	−	+	−	*Corynebacterium xerosis/Corynebacterium minutissimum/Corynebacterium kutscheri*
4.	Beach soil 2%	+	Rod	A	A	−	−	−	−	−	+	−	+	+	+	−	+	+	−	+	+	*Vibrio metschnikovii*
5.	Beach soil 4%	+	Rod	A	A	−	−	−	−	−	−	−	+	+	+	−	+	+	−	+	−	*Vibrio metschnikovii*
6.	Beach soil 6%	+	Rod	A	A	−	−	−	−	−	−	−	−	+	−	−	+	+	−	+	+	*Volucribacter psittacicida*
7.	Land soil 2%	+	Rod	K	A	−	−	−	−	−	−	−	−	−	+	+	−	−	−	−	+	*Aggregatibacter* (*Haemophilus*) *segnis*
8.	Land soil 4%	+	Rod	K	A	−	−	−	−	−	−	−	+	−	+	+	−	+	−	+	+	*Aggregatibacter* (*Haemophilus*) *segnis*
9.	Land soil 6%	+	Rod	A	K	−	−	−	−	−	−	−	+	−	+	−	−	+	−	+	+	*Clostridium innocuum/Clostridium spiroforme*

*K* alkaline reaction, *A* acidic reaction, *+* positive reaction, *−* negative reaction

In catalase test, hydrogen peroxide was used and broken down to water and oxygen. Patenga 2, 6% and Beach soil 2% were positive. Oxidase test resulted as negative for all (Table 2). Only Land soil 2 and 4% was reduced nitrate by the change of color (red) (Table 2). Most of the samples were responsive to TSI test. Both slant and butt became yellow for Patenga 2%, Beach soil 2, 4 and 6%. The only slant was acidic for Land soil 2 and 4% and Land soil 6% for the butt. Patenga 2%, Beach soil 2, 4 and 6% recorded as MR test positive. Patenga 4, 6%, Beach soil 2, 4%, Land soil 2, 4, and 6% found VP test positive because the color did not change to pink. All negative results recorded at MIU test, except non-motile Patenga 6% sample was urease positive. Simmon’s citrate test showed a positive result for Beach soil 2, 4% and land soil 4, 6%. Land soil 4, 6%, Patenga area 2, 4, 6%, Beach soil 2, 4, and 6% found positive for sucrose metabolism. No positive result observed for lactose fermentation. All the sample result was positive for dextrose fermentation, except Land soil 2%. Patenga area 2% and Beach soil 4% produced gas in fermentation. Patenga area 4% and Beach soil 2, 4, 6% hydrolyzed starch. Patenga 2, 4%, Beach soil 2, 6%, Land soil 2, 4 and 6% showed the ability to grow at 6.5% NaCl solution.

Finally, the test results were analyzed in ABIS online software [28], which is used to analyze the genus of the organism. Based on the morphology characteristics, Biochemical test and ABIS software the following results were predicted as Patenga 2%—*Brevibacillus agri;* Patenga 4%—*Pantoea stewartii* subsp. *stewartii;* Patenga 6%—*Corynebacterium xerosis/Corynebacterium minutissimum/Corynebacterium kutscheri;* Beach soil 2%—*Vibrio metschnikovii;* Beach soil 4%—*Vibrio metschnikovii;* Beach soil 6%—*Volucribacter psittacicida;* Land soil 2%—*Aggregatibacter* (*Haemophilus*) *segnis;* Land soil 4%—*Aggregatibacter* (*Haemophilus*) *segnis;* Land soil 6%—*Clostridium innocuum/Clostridium spiroforme*.

### Discussion

In this study, three soil samples were collected from the coastal area of Chittagong [29–31]. The isolated bacteria from the samples had successfully survived in a limited range of salinities and no halophilism detected. All the isolates showed tolerance to 2% (w/v), 4% (w/v) and 6% (w/v) of NaCl (Table 1). The dilution factor was inversely proportional to the number of colonies. The study divulged the abundance of gram positive bacteria (Table 2). The isolates from Patenga area (2, 6%) and Beach (2%) showed catalase activity and all the isolates showed negative results for oxidase activity, indole production, lactose and motility. Aerobic and facultative aerobes exhibit oxidase activity whereas *Enterobacteriaceae* are oxidase negative. This provided the evidence of *Enterobacteriaceae* in the samples. In the MIU test, only the isolates extracted from Patenga area that is 6% (w/v) NaCl tolerant showed urease positive. In addition to this, all the isolates provided positive results for dextrose fermentation. *Vibrio*s were predominating in the investigated soil along with *Corynebacterium, Clostridium* and *Enterobacteriaceae*.

## Limitations

Extreme halotolerant species as *Halomonas elongata CHR63*, *Thioalkalivibrio versutus* or *Sporosarcina pasteurii* wasn’t detected in the study [32]. The future prospect can be the plantation of transgenic plants in the coastal area for better agricultural use. The research also provides insights for adaption and use of soil microorganisms as natural fertilizers after natural calamity struck the coastal region. Before that, the success of this investigation is not properly accomplished.

## Abbreviations

MIU: motility indole urease test
et al.: and others
NA: nutrient agar
Mg: milligram
sp.: species
mL: milliliter
TSI: triple sugar iodine
MR: methyl red
VP: Voges Proskauer
w/v: weight by volume
g/L: gram per liter
